# Neighborhood Incarceration Rates and Adverse Birth Outcomes in New York City, 2010-2014

**DOI:** 10.1001/jamanetworkopen.2023.6173

**Published:** 2023-03-31

**Authors:** Louisa W. Holaday, Destiny G. Tolliver, Tiana Moore, Keitra Thompson, Emily A. Wang

**Affiliations:** 1 Division of General Internal Medicine, Department of Medicine, Icahn School of Medicine at Mount Sinai, New York, New York; 2 Institute for Health Equity Research, Icahn School of Medicine at Mount Sinai, New York, New York; 3 SEICHE Center for Health and Justice, Yale University, New Haven, Connecticut; 4 National Clinician Scholars Program, Yale University, New Haven, Connecticut; 5 Center for Vulnerable Populations, University of California, San Francisco; 6 Department of Social and Behavioral Sciences, Yale School of Public Health. Yale University, New Haven, Connecticut; 7 Section of General Medicine, Department of Internal Medicine, Yale School of Medicine, Yale University, New Haven, Connecticut

## Abstract

**Importance:**

The US has high rates of adverse birth outcomes, with substantial racial disparities augmented by stress and neighborhood disadvantage. Black people are more likely to live in neighborhoods with high rates of incarceration, which is a source of both stress and neighborhood disadvantage and, thus, may contribute to adverse birth outcomes.

**Objective:**

To determine whether neighborhoods with high incarceration rates also have higher rates of adverse birth outcomes compared with neighborhoods with lower rates.

**Design, Setting, and Participants:**

This cross-sectional study used publicly available data from the New York City Department of Health (2010-2014). Censored Poisson regression, with the US Census tract as the unit of analysis, was used to examine the association of neighborhood incarceration rate and birth outcomes. Multivariable models included percentage of births aggregated to the Census tract by maternal factors (age, parity, singleton vs multiple birth, insurance, and race) and neighborhood factors (poverty, education, and violent crime). Analyses were performed between May 2021 and October 2022.

**Exposure:**

Neighborhood incarceration rate, categorized into quintiles.

**Main Outcomes and Measures:**

The primary outcome was the incidence rate ratio (IRR) of preterm birth and low birth weight. Secondary outcomes were IRRs of very preterm birth, extremely preterm birth, and very low birth weight. Hypotheses were formulated before data collection.

**Results:**

Among 2061 Census tracts with 562 339 births, incarceration rates varied from 0 to 4545 people incarcerated per 100 000, and high-incarceration neighborhoods had more residents of Black race (54.00% vs 1.90%), living in poverty (32.30% vs 10.00%), and without a general educational development equivalent (28.00% vs 12.00%) compared with low-incarceration neighborhoods. In fully adjusted models, high-incarceration neighborhoods had a 13% higher IRR of preterm birth (IRR, 1.13; 95% CI, 1.08-1.18), 45% higher IRR of very preterm birth (IRR, 1.45; 95% CI, 1.24-1.71), 125% higher IRR of extremely preterm birth (IRR, 2.25; 95% CI, 1.59-3.18), 10% higher IRR of low birth weight (IRR, 1.10; 95% CI, 1.05-1.16), and 52% higher IRR of very low birth weight compared with low-incarceration neighborhoods (IRR, 1.52; 95% CI, 1.28-1.81).

**Conclusions and Relevance:**

Neighborhood incarceration rate was positively associated with adverse birth outcomes, particularly those associated with infant mortality. Black people were significantly more likely to live in high-incarceration neighborhoods, suggesting that mass incarceration may contribute to racial disparities in birth outcomes.

## Key Points

**Question:**

Do neighborhoods with high rates of incarceration have higher rates of adverse birth outcomes compared with neighborhoods with low rates of incarceration after adjusting for aggregated maternal and other neighborhood factors?

**Findings:**

In this cross-sectional study of 2061 US Census tracts in New York City with 562 339 births, fully adjusted models demonstrated a 13% higher incidence rate ratio of preterm birth and 10% higher incidence rate ratio of low birth weight in neighborhoods with high rates of incarceration compared with those with low rates of incarceration.

**Meaning:**

These findings suggest that living in a neighborhood with high rates of incarceration may contribute to adverse birth outcomes, and these neighborhoods warrant investment and further study.

## Teaser

This cross-sectional study examines whether neighborhoods with high incarceration rates also have higher rates of adverse birth outcomes compared with neighborhoods with lower incarceration rates.

## Introduction

The US has high infant mortality rates, ranking 34 of 38 among Organisation for Economic Co-operation and Development nations in 2019.^1^ Infant mortality is most commonly associated with preterm birth and low birth weight,^2^ with earlier preterm and lower weight newborns having higher risk of death.^3,4,5^ Rates of infant mortality have substantial disparities by race and socioeconomic status, with Black and low-income people most likely to have adverse birth outcomes.^6,7,8,9^ These disparities are associated with both individual-level socioeconomic factors and structural determinants of health that function at a neighborhood and societal level.^8,10,11,12,13,14,15^ A recent consensus statement on racial disparities on preterm birth highlighted stress and neighborhood disadvantage as likely factors.^10^

One structural determinant of health that may contribute to adverse birth outcomes through these pathways is mass incarceration.^16,17^ The US incarcerates more of its population than any other country in the world,^18^ yet neighborhood rates of incarceration are highly variable. Even within the same county, 2 neighborhoods may have incarceration rates as different as 0 people and 4000 people incarcerated per 100 000 population.^19,20,21^ People living in high-incarceration neighborhoods experience increased stress, disruption of family and social ties, and loss of financial and social resources and collective power.^18,21,22^ Living in a high-incarceration neighborhood is independently associated with worse mental health^23^ and an over 2-year lower life expectancy at birth,^19^ even after adjusting for neighborhood-level factors, including poverty, education, racial demographics (as a proxy for structural racism), violent crime, and population density.^19^ Thus, the indirect effects of mass incarceration on nonincarcerated individuals living in high-incarceration neighborhoods could influence birth outcomes through neighborhood disadvantage, a more stressful environment for pregnant people, and decreased availability of social or economic resources.^10,24,25^ Importantly, the same populations with higher rates of adverse birth outcomes—low-income and Black individuals in particular—are more likely to live in high-incarceration neighborhoods.^19,20,26^

Prior work has found associations between county-level rates of incarceration and birth outcomes^27,28^ and an association with state-level incarceration rate and infant mortality.^16,29^ However, people’s social networks and neighborhood resources may be more accurately reflected at a smaller geographic level. One study^30^ did examine the association of incarceration rates and preterm birth at the neighborhood level, but it was limited by a small sample and overall did not find a statistically significant association. In New York City (NYC), New York, racial disparities in birth outcomes and exposure to incarceration mirror national trends: non-Hispanic Black (hereafter, Black) people are most likely to have adverse birth outcomes and to live in high-incarceration neighborhoods.^19,31^ Thus, understanding how incarceration rates are associated with birth outcomes in NYC may be informative for policy makers both locally and nationally, particularly given ongoing conversations around decarceration, bail reform, and the close of NYC’s largest jail on Rikers Island.^32,33^

The goal of this study was to examine the association of neighborhood incarceration rates with adverse birth outcomes. Our primary outcomes were rates of preterm birth and low birth weight; secondary outcomes were rates of very preterm birth, extremely preterm birth, and very low birth weight. We hypothesized that neighborhoods with higher rates of incarceration would have higher incidence rate ratios (IRRs) of all adverse birth outcomes, particularly more negative birth outcomes.

## Methods

We conducted a cross-sectional study examining the association of neighborhood incarceration rates with adverse birth outcomes using data aggregated to the Census tract level. We used the Census tract as a stand-in for neighborhood, because Census tract boundaries are determined by the US Census Bureau in conjunction with cities and counties with the goal of reflecting neighborhoods.^34^ Our study follows Strengthening the Reporting of Observational Studies in Epidemiology (STROBE) reporting guideline^35^ and did not require review by the Yale University institutional review board or informed consent because data were publicly available, in accordance with 45 CFR §46.

### Independent Variable of Interest

Incarceration rate data were provided at the aggregate level by the Prison Policy Initiative and include 83.1% of all individuals incarcerated in New York state prisons at the time of the 2010 Census, excluding those who did not provide a prior address that could be geocoded to a Census tract in New York.^36^ State prisons make up the largest share of currently incarcerated individuals. Prisons house individuals convicted of a crime with a relatively longer sentence (typically at least 1 year), whereas jails primarily house those who are pretrial (>80% of the jail population) and also individuals postconviction with a relatively short sentence (typically <1 year).^37^ We modeled incarceration rate as a categorical variable, because it was significant as a quadratic variable in association with all birth outcomes. We used quintiles based on findings in our prior article^19^ showing that incarceration rate and life expectancy did not have a linear association, and the association was better reflected categorizing incarceration rate into quintiles.

### Dependent Variables

Birth outcome data were provided by the NYC Department of Health (NYCDOH) aggregated by Census tract from January 1, 2010, to December 31, 2014.^38^ Tracts with fewer than 50 births over this 5-year period were excluded because of NYCDOH data reporting practices. We examined 3 categories of preterm birth: all preterm births, defined as prior to 37 weeks’ gestation; very preterm births, defined as from 28 to 32 weeks’ gestation; and extremely preterm births, defined as prior to 28 weeks’ gestation. Extremely preterm newborns had a greater than 30% rate of death during this time period.^3^ We examined 2 categories of low birth weight: all low-birth-weight newborns, defined as less than 2500 g, and very-low-birth-weight newborns, defined as less than 1500 g. We modeled all outcomes as count data. The NYCDOH left-censors all cells with a count below 5, meaning that Census tracts with between 0 and 4 births in a given category over the 5-year data period were censored.

### Covariates

We included individual-level and neighborhood-level factors associated with adverse birth outcomes as covariates, aggregated at the Census tract level. From the NYCDOH birth data, we included aggregated data on the percentage of births to those younger than 18 and older than 40 years,^39^ with 2 or more newborns,^40^ and that were first newborns.^41^ For censored tracts, we substituted 0. To account for possible confounding due to both higher rates of adverse birth outcomes among Black people and the higher percentage of Black residents in high-incarceration neighborhoods,^10,18,26,31,42^ we adjusted for the percentage of births to Black people. Race and ethnicity data were provided by the NYCDOH on the basis of birth certificates completed by the birthing person. For censored tracts, we substituted the neighborhood percentage of Black residents. Because NYCDOH data did not include insurance status, we used tract-level insurance rates among women aged 18 to 45 years from the US Census American Community Survey (2010-2014), modeled as a continuous variable.^43^ As markers of neighborhood disadvantage, we included poverty rate and percentage of the population without a general educational development (GED) equivalent.^43^ To adjust for the possible confounding of living in a high-crime area and increased police contact, both of which may increase stress,^19,20,44^ we included violent crime rate. Violent crime rates were derived through geocoding of NYC Police Department data from 2010,^45^ described in our prior study.^19^ We did not include the racial demographics of the neighborhood as a proxy for exposure to structural racism,^46^ a commonly included variable in studies examining population health effects of incarceration, because of the near-perfect correlation (correlation coefficient, 0.98) with the racial demographics of the birthing person, which was already a covariate in the model.

We tested all neighborhood covariates (poverty rate, percentage without a GED equivalent, and violent crime rate) as quadratic terms to determine whether to include these as continuous or categorical variables in our multivariable models. Poverty and violent crime were consistently significant as quadratic terms and so were included as quintiles for ease of interpretation; education was consistently not significant as a quadratic term, so it was included as a continuous variable. One Census tract was missing violent crime data, so full models did not include this Census tract.

### Statistical Analysis

First, we performed univariate analyses to describe our sample and compared demographic variables across quintiles of incarceration. We used the Kruskal-Wallis test of difference between mean ranks across groups because all demographic variables were nonnormally distributed. To account for the censoring in the NYCDOH data, we modeled births data using censored Poisson regression, which is a method available in Stata that accounts mathematically for lower-limit censoring. Unadjusted models included only incarceration quintile and the outcome of interest. We constructed multivariable models for each birth outcome of interest using nested models that adjusted first for individual factors aggregated at the Census tract level (ie, age, number of newborns, and parity), then added insurance, next neighborhood disadvantage factors, and finally the percentage of births to Black people. We performed 2 sensitivity analyses. One modeled incarceration rate as deciles instead of quintiles, and the second substituted a count of 4 instead of 0 for all censored covariates. Analyses were performed between May 2021 and October 2022. Statistical tests were 2-tailed, and statistical significance was set at *P* < .05. Analyses were performed using Stata statistical software version 16.0 (StataCorp).

## Results

Our data set included 2061 Census tracts in New York City, excluding 88 with fewer than 50 births in the 5-year period per NYCDOH reporting practices, totaling 562 339 births, which is 91.6% of the total births in NYC from 2010 to 2014 (613 592 births).^47^ Incarceration rates varied from 0 to 4545 people incarcerated per 100 000. The quintile of neighborhoods with the highest incarceration rate had a median (IQR) of 698 (571-902) people incarcerated per 100 000, whereas the lowest-incarceration quintile had a median (IQR) of 19 (0-35) people incarcerated per 100 000. Neighborhoods with the most vs the least incarceration differed, including having more residents who were Black (median [IQR], 54.00% [35.10%-75.80%] vs 1.90% [0.80%-5.00%]), living in poverty (median [IQR], 32.30% [23.70%-41.90%] vs 10.00% [5.90%-17.40%]), and without a GED equivalent (median [IQR], 28.00% [20.00%-38.00%] vs 12.00% [6.00%-19.00%]) (Table 1).

**Table 1.** Neighborhood Characteristics Across Quintiles of Incarceration

Characteristic	Census tracts, median (IQR), % of residents					*P* value^a^
	First quintile	Second quintile	Third quintile	Fourth quintile	Fifth quintile	
Incarceration rate, No. of residents/100 000	19 (0-35)	75 (60-94)	151 (129-181)	339 (283-399)	698 (571-902)	<.001
No general educational development or equivalent	12.00 (6.00-19.00)	13.00 (8.00-21.00)	18.00 (12.00-27.00)	21.00 (15.00-32.00)	28.00 (20.00-38.00)	<.001
Poverty rate	10.00 (5.90-17.40)	12.00 (7.60-18.35)	15.00 (10.20-21.90)	21.30 (14.05-30.20)	32.30 (23.70-41.90)	<.001
Race and ethnicity^b^						
American Indian/Alaska Native	0.0 (0.0-0.2)	0.0 (0.0-0.3)	0.0 (0.0-0.5)	0.0 (0.0-0.5)	0.0 (0.0-0.6)	<.001
Asian	13.40 (5.10-29.40)	13.10 (5.50-27.50)	11.30 (4.70-24.20)	3.40 (0.90-8.05)	1.90 (0.40-4.70)	<.001
Black	1.90 (0.80-5.00)	3.35 (1.40-8.15)	7.30 (3.00-22.40)	37.95 (20.35-77.25)	54.00 (35.10-75.80)	<.001
Hispanic	10.30 (6.20-19.10)	13.55 (8.30-23.60)	20.20 (10.10-37.90)	28.65 (11.65-62.60)	35.00 (15.10-59.70)	<.001
Non-Hispanic White	64.10 (43.90-81.40)	54.70 (25.15-73.95)	27.80 (5.80-59.30)	4.85 (1.60-18.45)	3.10 (0.90-10.90)	<.001
Women aged 18-45 y without insurance	7.20 (3.80-12.30)	9.25 (5.30-14.50)	11.50 (6.70-16.60)	12.10 (8.85-15.80)	11.40 (8.10-14.80)	<.001
Violent crime rate, No./1000 residents	1.31 (0.67-2.65)	2.07 (1.04-3.34)	3.11 (1.70-5.21)	5.39 (3.51-8.12)	7.99 (5.65-10.90	<.001

^a^ Difference across groups were tested using the Kruskal-Wallis test of difference between mean ranks across groups.

^b^ Neighborhood racial demographics do not add up to 100% because they are group medians, and do not include people who identified as more than 1 race.

### Models

#### Preterm Birth

**Figure 1.**
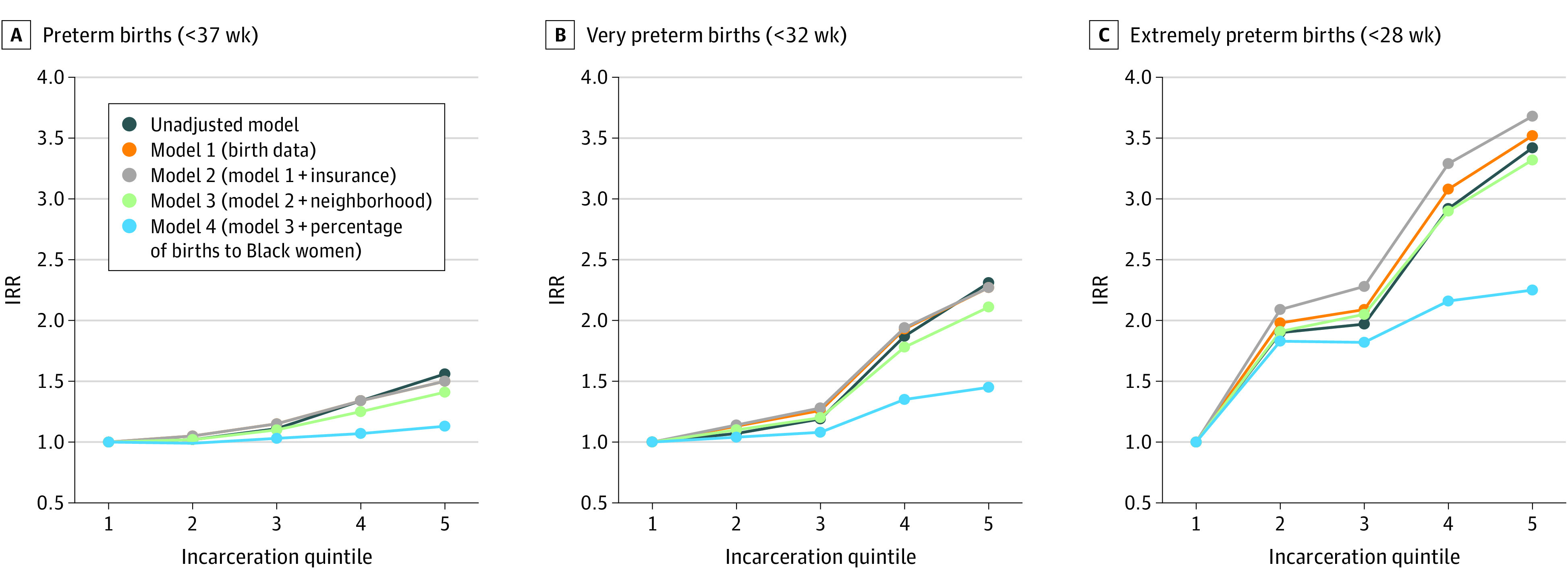
Association of Neighborhood Incarceration Rate and Preterm Birth Graphs show incidence rate ratios (IRRs) of preterm birth (A), very preterm birth (B), and extremely preterm birth (C) by neighborhood incarceration quintile. The lowest incarceration quintile serves as the reference with an IRR of 1.0 in all models.

In all models, as neighborhood incarceration rate increased, there was an increased IRR of preterm birth, with the greatest difference between the first and fifth quintile of incarceration. As hypothesized, worse birth outcomes were more strongly associated with high incarceration rates. In unadjusted models, the neighborhoods with the highest incarceration rates had a 56% higher IRR of preterm birth (IRR, 1.56; 95% CI, 1.52-1.61; *P* < .001), a 131% higher IRR of very preterm birth (IRR, 2.31; 95% CI, 2.08-2.56; *P* < .001), and 242% higher IRR of extremely preterm birth (IRR, 3.42; 95% CI, 2.63-4.44; *P* < .001), compared with neighborhoods with the least incarceration. After adjusting for all covariates, these associations persisted, including a 13% higher IRR of preterm birth (IRR, 1.13; 95% CI, 1.08-1.18; *P* < .001), 45% higher IRR of very preterm birth (IRR, 1.45; 95% CI, 1.24-1.71; *P* < .001), and a 125% higher IRR of extremely preterm birth (IRR, 2.25; 95% CI, 1.59-3.18; *P* < .001). For all models, the covariate that explained most of the difference between unadjusted and adjusted models was the percentage of births to Black people (Figure 1 and Table 2).

**Table 2.** Association of Neighborhood Incarceration Rate and Preterm Birth

Preterm birth category and incarceration quintile	IRR (95% CI)				
	Unadjusted model	Model 1^a^	Model 2^b^	Model 3^c^	Model 4^d^
All preterm births (<37 wk)					
1	1 [Reference]	1 [Reference]	1 [Reference]	1 [Reference]	1 [Reference]
2	1.02 (0.98-1.05)	1.05 (1.01-1.09)	1.05 (1.01-1.08)	1.02 (0.98-1.06)	0.99 (0.96-1.03)
3	1.11 (1.08-1.15)	1.15 (1.11-1.19)	1.15 (1.11-1.19)	1.10 (1.06-1.14)	1.03 (0.99-1.07)
4	1.34 (1.30-1.38)	1.34 (1.30-1.39)	1.34 (1.29-1.38)	1.25 (1.20-1.30)	1.07 (1.02-1.11)
5	1.56 (1.52-1.61)	1.50 (1.45-1.55)	1.50 (1.45-1.55)	1.41 (1.35-1.47)	1.13 (1.08-1.18)
* P *value^e^	<.001	<.001	<.001	<.001	<.001
Very preterm births (<32 wk)					
1	1 [Reference]	1 [Reference]	1 [Reference]	1 [Reference]	1 [Reference]
2	1.07 (0.94-1.22)	1.13 (0.99-1.29)	1.14 (0.99-1.30)	1.10 (0.96-1.26)	1.04 (0.90-1.20)
3	1.19 (1.05-1.35)	1.26 (1.11-1.44)	1.28 (1.12-1.45)	1.20 (1.05-1.39)	1.08 (0.93-1.25)
4	1.87 (1.68-2.08)	1.93 (1.72-2.17)	1.94 (1.73-2.18)	1.78 (1.54-2.05)	1.35 (1.16-1.57)
5	2.31 (2.08-2.56)	2.27 (2.01-2.55)	2.27 (2.02-2.56)	2.11 (1.82-2.46)	1.45 (1.24-1.71)
* P *value^e^	<.001	<.001	<.001	<.001	<.001
Extremely preterm births (<28 wk)					
1	1 [Reference]	1 [Reference]	1 [Reference]	1 [Reference]	1 [Reference]
2	1.90 (1.42-2.54)	1.98 (1.48-2.66)	2.09 (1.55-2.82)	1.91 (1.40-2.61)	1.83 (1.34-2.51)
3	1.97 (1.48-2.62)	2.09 (1.56-2.78)	2.28 (1.70-3.07)	2.05 (1.49-2.83)	1.82 (1.32-2.52)
4	2.92 (2.24-3.81)	3.08 (2.35-4.06)	3.29 (2.48-4.36)	2.90 (2.09-4.01)	2.16 (1.55-3.02)
5	3.42 (2.63-4.44)	3.52 (2.67-4.64)	3.68 (2.77-4.89)	3.32 (2.38-4.64)	2.25 (1.59-3.18)
* P *value^e^	<.001	<.001	<.001	<.001	<.001

Abbreviation: IRR, incidence rate ratio.

^a^ Model 1 includes percentage of births to people younger than 18 or older than 40 years, percentage of births with multiples (eg, twins or triplets), and parity of birthing person.

^b^ Model 2 includes model 1 variables plus the percentage of women aged 18 to 45 years without insurance.

^c^ Model 3 includes model 2 variables plus neighborhood poverty, education, and violent crime rates.

^d^ Model 4 includes model 3 variables plus the percentage of births to non-Hispanic Black people.

^e^ *P *value is for joint test of association of incarceration rate and outcome of interest.

#### Low Birth Weight

**Figure 2.**
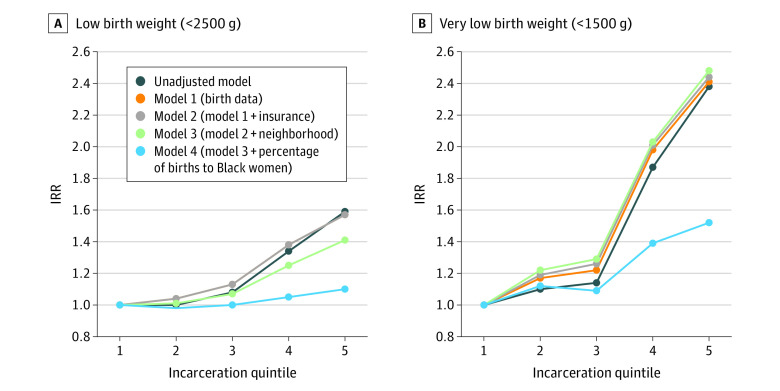
Association of Neighborhood Incarceration Rate and Low Birth Weight Graphs show incidence rate ratios (IRRs) of low birth weight (A) and very low birth weight (B) by neighborhood incarceration quintile. The lowest incarceration quintile serves as the reference with an IRR of 1.0 in all models.

In all models, as neighborhood incarceration rate increased, there was an increased IRR of low birth weight, with the greatest difference between the first and fifth quintile of incarceration. In unadjusted models, the neighborhoods with the highest incarceration rates had a 59% higher IRR of low birth weight (IRR, 1.59; 95% CI, 1.54-1.64; *P* < .001) and a 138% higher IRR of very low birth weight (IRR, 2.38; 95% CI, 2.12-2.68; *P* < .001), compared with neighborhoods with the lowest incarceration rates. These findings persisted after adjusting for all covariates, including a 10% higher IRR of low birth weight (IRR, 1.10; 95% CI, 1.05-1.16; *P* < .001), and 52% higher IRR of very low birth weight (IRR, 1.52; 95% CI, 1.28-1.81; *P* < .001). Here too, for all models, the covariate that explained most of the difference between unadjusted and adjusted models was the percentage of births to Black people (Figure 2 and Table 3). Findings in all models were robust to our sensitivity analyses.

**Table 3.** Association of Neighborhood Incarceration Rate and Low Birth Weight

Birth weight category and incarceration quintile	IRR (95% CI)				
	Unadjusted model	Model 1^a^	Model 2^b^	Model 3^c^	Model 4^d^
All low-birth-weight newborns (<2500 g)					
1	1 [Reference]	1 [Reference]	1 [Reference]	1 [Reference]	1 [Reference]
2	1.00 (0.96-1.04)	1.04 (1.00-1.08)	1.04 (1.00-1.08)	1.01 (0.97-1.05)	0.98 (0.95-1.02)
3	1.08 (1.04-1.11)	1.13 (1.09-1.17)	1.13 (1.09-1.17)	1.07 (1.03-1.11)	1.00 (0.96-1.04)
4	1.34 (1.30-1.39)	1.38 (1.33-1.42)	1.38 (1.33-1.43)	1.25 (1.20-1.30)	1.05 (1.00-1.09)
5	1.59 (1.54-1.64)	1.57 (1.51-1.63)	1.57 (1.51-1.63)	1.41 (1.35-1.48)	1.10 (1.05-1.16)
* P *value^e^	<.001	<.001	<.001	<.001	<.001
Very-low-birth-weight newborns (<1500 g)					
1	1 [Reference]	1 [Reference]	1 [Reference]	1 [Reference]	1 [Reference]
2	1.10 (0.95-1.29)	1.17 (1.01-1.37)	1.19 (1.02-1.39)	1.22 (1.04-1.42)	1.12 (0.95-1.31)
3	1.14 (0.98-1.32)	1.22 (1.05-1.42)	1.26 (1.08-1.47)	1.29 (1.11-1.51)	1.09 (0.93-1.28)
4	1.87 (1.65-2.11)	1.98 (1.73-2.26)	2.01 (1.76-2.30)	2.03 (1.76-2.35)	1.39 (1.18-1.63)
5	2.38 (2.12-2.68)	2.41 (2.11-2.76)	2.44 (2.13-2.79)	2.48 (2.13-2.88)	1.52 (1.28-1.81)
* P *value^e^	<.001	<.001	<.001	<.001	<.001

Abbreviation: IRR, incidence rate ratio.

^a^ Model 1 includes percentage of births to people younger than 18 or older than 40 years, percentage of births with multiples (eg, twins or triplets), and parity of birthing person.

^b^ Model 2 includes model 1 variables plus the percentage of women aged 18 to 45 years without insurance.

^c^ Model 3 includes model 2 variables plus neighborhood poverty, education, and violent crime rates.

^d^ Model 4 includes model 3 variables plus the percentage of births to non-Hispanic Black people.

^e^ *P* value is for joint test of association of incarceration rate and outcome of interest.

## Discussion

In this cross-sectional study, we found that neighborhoods in NYC with high rates of incarceration had significantly greater rates of adverse birth outcomes compared with neighborhoods with lower incarceration rates, even after controlling for potential confounders aggregated at the Census tract level. Our findings expand on prior work observed at the county level.^27,28^ Here, we used a smaller geographic area to approximate neighborhood, include all racial groups, and examine multiple adverse birth outcomes. We found a 1.5 to 4 times higher IRR for overall preterm birth in high-incarceration neighborhoods compared with what had previously been reported for high-incarceration counties.^27,28^ This finding suggests that population-level health impacts of incarceration may be best measured and understood at a neighborhood level.

Furthermore, we found that extremely preterm birth had the greatest association with neighborhood incarceration rate, suggesting that, consistent with prior work at the state level,^16,29^ mass incarceration may contribute to persistently high rates of infant mortality in the US. As the relative rates of infant mortality in the US compared with peer nations have increased in step with the rates of incarceration in the last 50 years, further exploration of mass incarceration as a contributing factor to infant mortality is warranted.^48^

The association between incarceration rates and birth outcomes persisted even after adjusting for the percentage of births to Black people, suggesting that living in a high-incarceration neighborhood is itself a factor associated with increased risk of adverse birth outcomes and not simply a proxy for neighborhood racial demographics and known racial disparities in birth outcomes. Our work extends prior county-level work, which only examined birth outcomes among Black and White birthing people. Our data suggest that living in a high-incarceration neighborhood is associated with adverse birth outcomes across racial groups. However, since Black people are significantly more likely to live in high-incarceration neighborhoods, mass incarceration may still directly contribute to racial disparities in birth outcomes and infant mortality.^17^ Our finding that the racial demographics of the birthing persons explains much, but not all, of the association between incarceration rate and birth outcomes underscores the need for a multifaceted approach to address structural racism at both the individual and community level to improve birth outcomes.

Adverse birth outcomes in neighborhoods with high rates of incarceration cannot be attributed only to newborns whose parent was incarcerated during gestation. The highest-incarceration neighborhoods had a median of less than 1% of the population incarcerated at a given time. Furthermore, a study^49^ using individual birth data in NYC from 2010 to 2016 found that only 0.9% of newborns had parental jail incarceration during gestation. That study found that paternal incarceration at some point during gestation was associated with a 34% to 39% higher risk of preterm birth or low birth weight, after adjusting for parental sociodemographics, maternal health behaviors, and health care access.^49^ Thus, if our findings were due to only increased adverse birth outcomes among those with a parent incarcerated during gestation, rather than factors associated with living in the neighborhood even for pregnant people with nonincarcerated partners, we would expect IRRs at least an order of magnitude lower than what we found. To be clear, our results suggest that living in a neighborhood with high rates of incarceration is associated with adverse birth outcomes even for newborns who did not have an incarcerated parent.

The causal mechanisms behind our findings warrant further study. Adverse birth outcomes in neighborhoods with high rates of incarceration are likely due to spillover effects of incarceration onto nonincarcerated individuals. Living in a neighborhood with high incarceration is associated with worse mental health,^23^ and increased stress among pregnant persons in these neighborhoods may contribute to adverse birth outcomes.^25^ Neighborhoods with high rates of incarceration also have less social cohesion and fewer financial and community resources, as policy dollars are spent on policing rather than community building.^21,22^ One recent study^50^ demonstrated improved access and adherence to prenatal care in counties with decreasing rates of prison admissions. Thus, prenatal care access may be lower in high-incarceration neighborhoods.

Our findings suggest a need to invest in communities that experience high rates of incarceration and to identify specific needs and barriers to care for pregnant people living there. Mass incarceration has had a profound health impact on communities, particularly communities of color, which needs to be addressed before more harm is done.^26^ Our work demonstrates that even those individuals who do not themselves experience incarceration are harmed by population-level exposure to it, including newborns. Policy makers in New York state considering changes to incarceration policy should consider these spillover effects. New York has relatively low rates of incarceration compared with the rest of the US, yet rates in New York are still almost triple those of our peer nations.^51^ Furthermore, the NYC neighborhoods with the highest rates of incarceration match the average rate in the US^51^ and underscore the need for research on the community-level effects of incarceration.

### Limitations

Our study has some limitations. Census tracts do not perfectly reflect lived neighborhoods and social networks, but these small geographic areas are the best representation in this data set. Our data include only those incarcerated at state prisons who provided an address that could be geocoded. We expect that those missing address data are more likely to come from high-incarceration neighborhoods. Neighborhoods with high rates of state prison incarceration are also likely to have high rates of jail and federal prison incarceration. Thus, our data likely underestimate the overall prevalence of incarceration in these neighborhoods, but accurately represent the neighborhood quintiles, which are relative rates, used in this study. Our data were limited because they were aggregated, so we could not stratify by race and ethnicity. This also limits the interpretation of the contribution of teen births, because most tracts had censored data on this variable, yet our overall findings were robust to sensitivity analyses substituting the upper and lower limits of the censored data. Future work with individual-level birth data would expand on our findings. Our data are limited by the remote time frame; however, these were the most recently available birth outcomes data from the NYCDOH, and incarceration rates have been stable. In addition, these data are cross-sectional and cannot definitively identify a causal relationship. However, our findings indicate that neighborhoods with high incarceration rates are vulnerable to negative health outcomes.

## Conclusions

In conclusion, pregnant people living in neighborhoods with high rates of incarceration are at higher risk of adverse birth outcomes, suggesting that mass incarceration may contribute to high rates of adverse birth outcomes and infant mortality in the US. Furthermore, because neighborhoods with high rates of incarceration have significantly higher proportions of Black residents, mass incarceration is a likely contributor to racial disparities in adverse birth outcomes and infant mortality.^17^ Such neighborhoods warrant increased support, investment, and further study.

## Notes

Supplement.
Data Sharing Statement
