# Supplementary material for: Neighborhood Incarceration Rates and Adverse Birth Outcomes in New York City, 2010-2014
Source: JAMA Netw Open. 2023 Mar 31;6(3):e236173. doi: 10.1001/jamanetworkopen.2023.6173 (PMC10066462; doi:10.1001/jamanetworkopen.2023.6173)
Supplement: Supplement. — Data Sharing Statement [file jamanetwopen-e236173-s001.pdf]

Data Sharing Statement

Holaday. Neighborhood Incarceration Rates and Adverse Birth Outcomes in New York City, 2010-2014. *JAMA Netw Open*. Published March 31, 2023.
doi:10.1001/jamanetworkopen.2023.6173

Data
Data available: Yes
Data types: Other (please specify)
Additional Information: Data are publicly available
How to access data: https://www1.nyc.gov/site/doh/data/data-sets/vital-statistics-birth-micro-sas-datasets.page
When available: With publication

Supporting Documents
Document types: None

Additional Information
Who can access the data: anyone requesting the data
Types of analyses: for any purpose
Mechanisms of data availability: without investigator support
